# The clathrin adaptor complex-1 and Rab12 regulate post-golgi trafficking of WT epidermal growth factor receptor (EGFR)

**DOI:** 10.1016/j.jbc.2023.102979

**Published:** 2023-02-04

**Authors:** Jinhui Wang, Pik Ki Lau, Chun Wa Li, Yusong Guo

**Affiliations:** 1 Division of Life Science and State Key Laboratory of Molecular Neuroscience, The Hong Kong University of Science and Technology, Hong Kong, China; 2 Hong Kong University of Science and Technology Shenzhen Research Institute, Shenzhen, China; 3 Southern Marine Science and Engineering Guangdong Laboratory (Guangzhou), Guangzhou, China

**Keywords:** cargo sorting, trans-Golgi network, EGFR, clathrin adaptor complex-1, Rab12, AP-1, adaptor complex-1, CT, cycle threshold, DAPI, 4′,6-diamidino-2-phenylindole, ER, endoplasmic reticulum, EGF, epidermal growth factor, EGFR, epidermal growth factor receptor, EGFP, enhanced GFP, FBS, fetal bovine serum, KD, knockdown, pEGFR, phosphorylated EGFR, RUSH, Retention Using Selective Hooks, RT-qPCR, quantitative reverse transcription RT-PCR, SBP, streptavidin binding protein, TGN, trans-Golgi network, WB, Western blot

## Abstract

The epidermal growth factor receptor (EGFR) plays important roles in cancer progression and is one of the major drug targets for targeted cancer therapy. Although fundamentally important, how newly synthesized EGFR is delivered to the cell surface to perform its cellular functions remains to be further investigated. In this study, we found using the approaches of gene knockout, siRNA knockdown, streptavidin pull-down, and co-immunoprecipitation assays that the clathrin adaptor complex-1 (AP-1) and Rab12 interact with EGFR and regulate the export of EGFR out of the *trans*-Golgi network (TGN). In addition, the tyrosine residue at the 998 position on human EGFR is critical to bind to AP-1, and this residue is important for TGN export of EGFR. We demonstrate that AP-1 and Rab12 are important for epidermal growth factor–induced phosphorylation of EGFR, cell elongation, and proliferation, suggesting that AP-1–mediated and Rab12-mediated post-Golgi trafficking is important for EGFR signaling. Moreover, TGN export of the constitutively activated mutant form of EGFR (EGFR^L858R^) is independent of AP-1 and Rab12. Our results reveal insights into the molecular mechanisms that mediate the TGN-to-cell surface delivery of EGFR and indicate that TGN export of WT EGFR and EGFR^L858R^ depends on different cellular factors.

The epidermal growth factor receptor (EGFR) is a cell surface-localized receptor for epidermal growth factor (EGF) and transforming growth factor-α. Upon ligand binding, EGFR activates downstream signaling pathways, leading to cell growth and proliferation. EGFR plays important roles in cancer progression, and it is one of the major drug targets for targeted cancer therapy (1). The function of WT EGFR relies on its localization on the cell surface, which is controlled by the endocytic and biosynthetic trafficking of EGFR (2, 3). Although significant progress has been achieved in understanding the endocytic trafficking of EGFR, the molecular mechanisms mediating the biosynthetic EGFR trafficking remain to be further investigated.

Newly synthesized EGFR follows the conventional steps in the secretory transport pathway to be delivered to the cell surface. After being synthesized from ribosomes, EGFR is first translocated into the endoplasmic reticulum (ER) membranes. The export of EGFR out of the ER depends on the COPII subunits, SEC24B, SEC24D, and SEC23B (3). Interestingly, prolonged EGF treatment has been reported to not only induce internalization and partial degradation of surface-located EGFR but also trigger an upregulation of the expression of these COPII subunits to promote surface delivery of EGFR (3). These analyses suggest that a feed-forward loop exists within the cell to adjust the efficiency of EGFR synthesis and transport to restore the level of EGFR on the cell surface (3).

The *trans*-Golgi network (TGN) is another important station in the secretory pathway. At the TGN, EGFR is packaged into transport vesicles, and these transport vesicles are transported along post-Golgi trafficking routes to the plasma membrane. Arf family proteins and cargo adaptors are key players that regulate protein sorting at the TGN (4). In *Caenorhabditis* elegans, ARF 1.2 and ARF-3 function with the clathrin adaptor complex-1 (AP-1) to regulate EGFR localization and signaling (5), but whether these factors regulate the trafficking of EGFR at the TGN or endosomes remains unclear. In humans, the cargo adaptor GGA2 interacts with EGFR and regulates EGFR trafficking in early endosomes/multivesicular bodies (6). Another study showed that AP-1 and GGA2 interact with EGFR in Rab11-positive recycling endosomes to regulate the retrieval of endocytosed EGFR, thereby sustaining the surface expression of EGFR (7). These analyses provide insights into post-Golgi EGFR trafficking that takes place at the endosomes; however, the molecular mechanisms regulating TGN export of EGFR are still poorly understood.

Many oncogenic mutations on EGFR have been identified in cancer cells. One of the most common mutations is a point mutation (L858R) in exon 21, which takes place in approximately 40% of lung cancer patients (8). EGFR L858R substitution lies in the activation loop of the EGFR kinase domain (9). Structural analysis indicates that the WT EGFR kinase domain adopts an inactive conformation. L858R substitution disrupts interactions that stabilize the inactive conformation, thereby locking the enzyme in a constitutively active conformation (9). In addition, L858R substitution facilitates EGFR dimerization to activate kinase activity (10). The structural changes of EGFR induced by L858R substitution indicate that EGFR^L858R^ may interact with novel cellular factors to regulate its intracellular trafficking. Currently, whether WT EGFR and EGFR^L858R^ utilize the same molecular machinery to regulate their intracellular trafficking to the cell surface remains unknown.

Here, we investigated the molecular mechanisms regulating post-Golgi trafficking of EGFR. Our study reveals two cellular factors, AP-1 and Rab12, that regulate the TGN export of WT EGFR, and these two factors are important for EGF-induced EGFR signaling. Moreover, we found that the TGN export of EGFR^L858R^ is independent of AP-1 and Rab12. These analyses provide novel insights into the biosynthetic trafficking of EGFR and indicate that post-Golgi trafficking of WT EGFR and EGFR^L858R^ depends on distinct cellular factors.

## Results

### AP-1 regulates TGN export of EGFR

**Figure 1:**
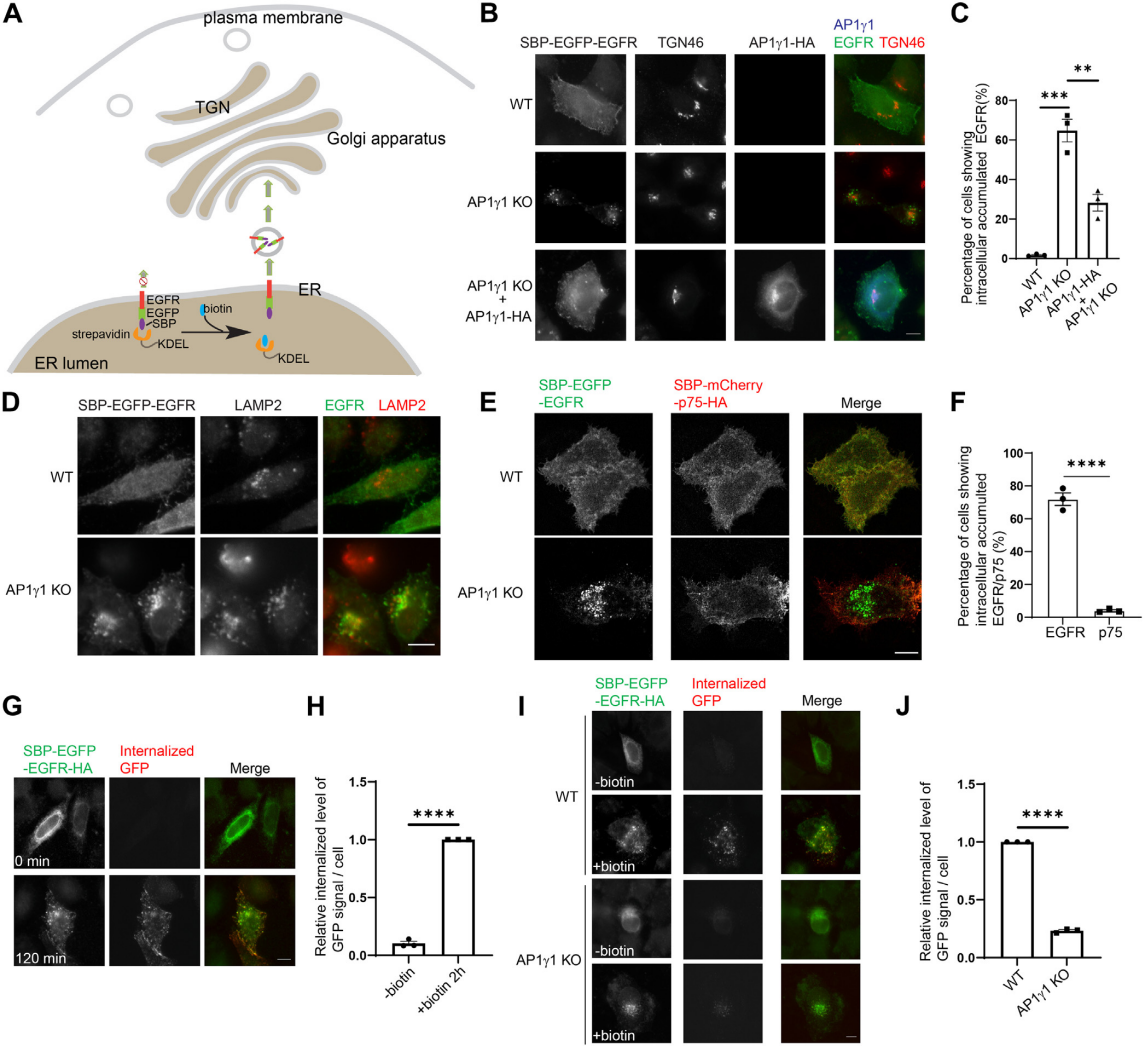
**AP-1 regulatesthe TGN-to-cell surface delivery of EGFR.***A*, a diagram demonstrating the RUSH transport assay. *B and D*, WT HeLa cells or AP1γ1 KO HeLa cells were transiently transfected with plasmids, encoding the indicated constructs. Day 1 after transfection, the cells were incubated with biotin for 2 h, and the localizations of the indicated proteins were analyzed by immunofluorescence. The size bar represents 10 μm. *C*, the percentage of cells showing intracellular accumulated EGFR in *B* was quantified (*n* = 3, mean ± SEM, over 100 cells were quantified in each experimental group). *E*, WT HeLa cells or AP1γ1 KO HeLa cells coexpressing SBP-EGFP-EGFR and SBP-mCherry-p75-HA were incubated with biotin for 2 h, and the localizations of the indicated proteins were analyzed by immunofluorescence. The size bar represents 10 μm. *F*, the percentage of cells showing intracellular accumulated EGFR or p75 in *E* was quantified (*n* = 3, mean ± SEM, over 100 cells were quantified in each experimental group). *G*–*J*, WT HeLa cells or AP1γ1 KO HeLa cells were transiently transfected with SBP-EGFP-EGFR-HA. Day 1 after transfection, rabbit anti-GFP antibodies were added to the medium in the presence or absence of biotin for 2 h. Then, internalized GFP antibodies were labeled by secondary antibodies against rabbit conjugated with 568 fluorophore. The size bar represents 10 μm. The ratio of internalized above-threshold fluorescent level of GFP signal over the above-threshold fluorescent level of total SBP-EGFP-EGFR-HA signal per cell was quantified (*H* and *J*, *n* = 3, mean ± SEM, over 15 cells were quantified in each experimental group). The quantification was normalized to the +biotin group (*H*) or to the WT group (*J*). ∗∗*p* < 0.01; ∗∗∗*p* < 0.001; ∗∗∗∗*p* < 0.0001. AP-1, adaptor complex-1; EGFR, epidermal growth factor receptor; EGFP, enhanced GFP; RUSH, Retention Using Selective Hooks; RT-qPCR, quantitative reverse transcription RT-PCR; SBP, streptavidin binding protein; TGN, trans-Golgi network.

AP-1 is a major TGN-localized cargo adaptor. To test whether AP-1 regulates TGN export of EGFR, we analyzed the trafficking of EGFR in HeLa cells knockout of the gamma subunit of AP-1 (AP1γ1) through a Retention Using Selective Hooks (RUSH) assay (11, 12). In the RUSH assay, EGFR was fused with enhanced GFP (EGFP) and streptavidin binding protein (SBP-EGFP-EGFR) and initially trapped in the ER by binding to luminal streptavidin fused with ER retention signal KDEL (Str-KDEL). The addition of biotin, which competes with SBP for binding to streptavidin, causes a synchronized release of cargo from the ER to downstream compartments (Fig. 1*A*). After biotin release for 2 h, SBP-EGFP-EGFR was transported from the ER to the plasma membrane in a majority of WT HeLa cells (Fig. 1*B*). At this time point, EGFR accumulated at intracellular punctate structures with no detectable surface pattern in around 60% of AP1γ1 KO HeLa cells. This percentage was significantly higher than that detected in WT HeLa cells (Fig. 1, *B* and *C*). The expression of an HA-tagged AP1γ1 (AP1γ1-HA) in AP1γ1 KO cells significantly decreased the percentage of cells showing accumulation of SBP-EGFP-EGFR at intracellular punctate structures (Fig. 1, *B* and *C*). Many of the intracellular accumulated EGFR colocalized with the lysosome marker, LAMP2 (Fig. 1*D*), suggesting that SBP-EGFP-EGFR was missorted to lysosomes in AP1γ1 KO cells. During this trafficking process, EGFR may be delivered from the TGN to the early endosomes or the recycle endosomes before reaching lysosomes in AP1γ1 KO cells. We then generated the RUSH construct of another plasma membrane–located protein, SBP-mCherry-p75-HA. EGFR and p75 are located on the basolateral and apical plasma membrane in polarized epithelial cells (13, 14), respectively. WT or AP1γ1 KO HeLa cells were cotransfected with plasmids encoding SBP-EGFP-EGFR or SBP-mCherry-p75-HA. We then analyzed the localizations of these proteins in cells coexpressing these two RUSH constructs. As the signal of mCherry was weak, we used antibodies against HA to label SBP-mCherry-p75-HA. We found that AP1γ1 KO causes defects in the surface delivery of SBP-EGFP-EGFR but not SBP-mCherry-p75-HA (Fig. 1, *E* and *F*). This result suggests that the surface delivery of SBP-mCherry-p75-HA is independent of AP-1.

We then performed a GFP antibody uptake assay to test whether the intracellularly accumulated RUSH construct of EGFR in AP1γ1 KO cells was delivered from the TGN along the biosynthetic anterograde pathway or delivered from the plasma membrane along the endocytic pathway. In this assay, SBP-EGFP-EGFR-HA delivered to the cell surface was labeled by rabbit anti-GFP antibodies detecting the extracellularly exposed EGFP tag (Fig. 1*G*). The fluorescent signal from the EGFP tag was used to label the total signal of SBP-EGFP-EGFR-HA (Fig. 1*G*). The immunofluorescence signal labeled by the internalized GFP antibodies was significantly increased in cells in the presence of biotin, suggesting that SBP-EGFP-EGFR-HA was delivered to cell surface after biotin treatment (Fig. 1, *G* and *H*). The knockout of AP1γ1 caused a significant reduction of the immunofluorescence signal labeled by the internalized GFP antibodies (Fig. 1, *I* and *J*), indicating that the depletion of AP1γ1 induced defects in the surface delivery of SBP-EGFP-EGFR-HA. We observed that SBP-EGFP-EGFR-HA showed a punctate localization pattern after biotin treatment (Fig. 1, *G* and *I*), which might be due to the enhancement of EGFR internalization or defects in surface retrieval after adding GFP antibodies. Live imaging analysis indicated that SBP-EGFP-EGFR-HA was delivered from the ER to the juxtanuclear Golgi area after biotin treatment. Subsequently, punctate structures of SBP-EGFP-EGFR-HA were detected during post-Golgi trafficking of SBP-EGFP-EGFR-HA, and many of these punctate structures were delivered to the cell periphery en route to the plasma membrane (Movie S1). By contrast, SBP-EGFP-EGFR-HA was highly accumulated at the intracellular area with no detectable surface-located pattern after biotin treatment in AP1γ1 KO HeLa cells (Movie S2). These analyses suggest that AP-1 regulates TGN-to-cell surface trafficking of EGFR.

### AP-1 interacts with the Y^998^RAL motif of EGFR to regulate its TGN export

**Figure 2:**
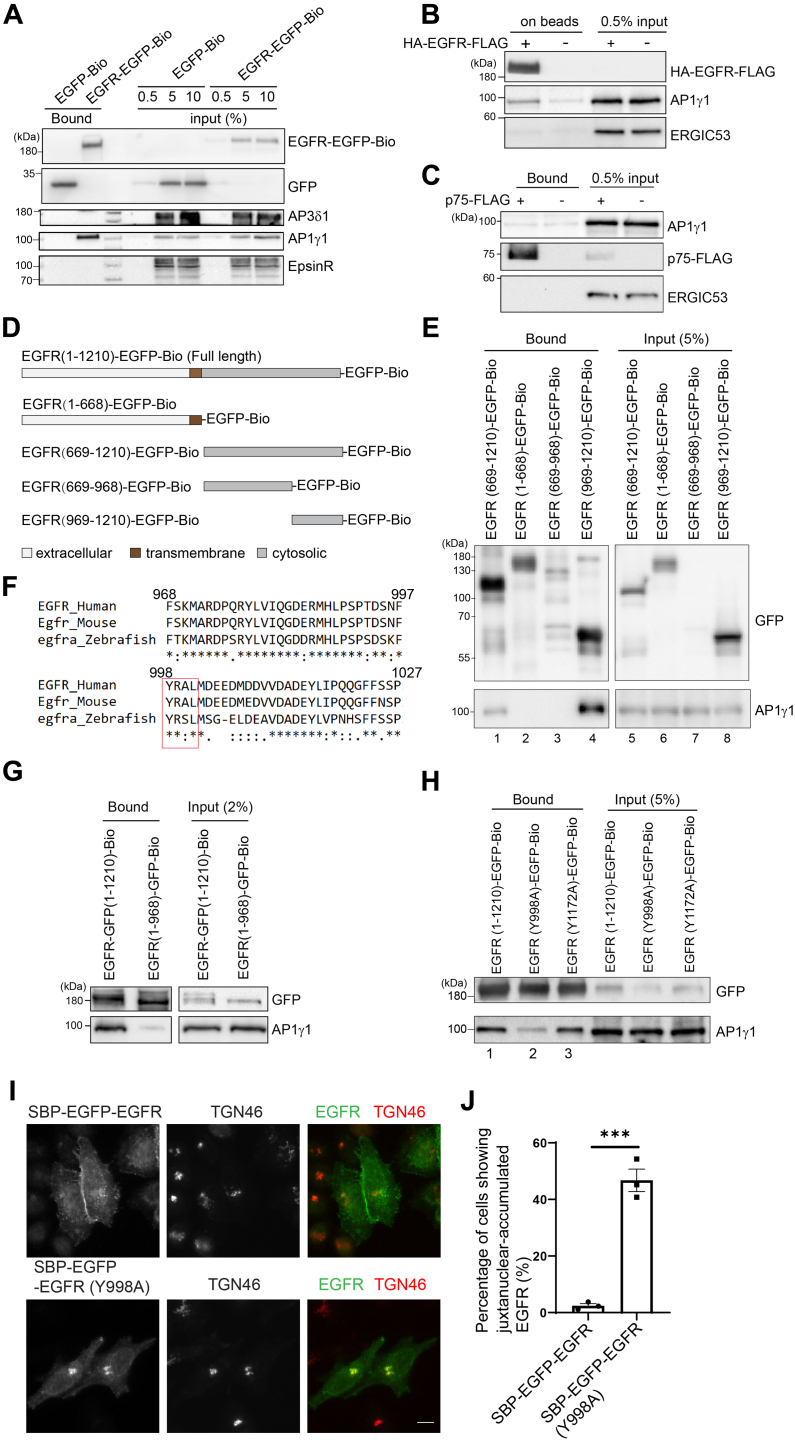
**AP-1 interacts with Y**^**998**^**RAL motif of EGFR to regulate TGN export of EGFR.***A*, HEK293T cells coexpressing HA-BirA with EGFR-EGFP-Bio or EGFP-Bio were harvested and incubated with streptavidin beads. The bound proteins were analyzed by immunoblotting. *B* and *C*, lysates from untransfected HEK293T cells, or HEK293T cells expressing HA-EGFR-FLAG (*B*) or p75-FLAG (*C*) were incubated with M2 agarose beads. The bound proteins were analyzed by immunoblotting. *D*, diagram showing the different EGFP-Bio-tagged EGFR fragments. *E*, *G*, and *H*, lysates from HEK293T cells coexpressing HA-BirA, and the indicated constructs were incubated with streptavidin beads. The bound proteins were analyzed by immunoblotting. *F*, sequence alignment of EGFR in the C-terminal tail region across different species. *I*, HeLa cells were transiently transfected with SBP-EGFP-EGFR or SBP-EGFP-EGFR^Y998A^. Day 1 after transfection, SBP-EGFP-EGFR or SBP-EGFP-EGFR^Y998A^ was released by adding biotin for 2 h. The localizations of the indicated proteins were analyzed by immunofluorescence. The size bar represents10 μm. *J*, the percentage of cells showing juxtanuclear-accumulated EGFR at the Golgi area was quantified (*n* = 3, mean ± SEM, over 100 cells were quantified in each experimental group). ∗∗∗*p* < 0.001. AP-1, adaptor complex-1; EGFR, epidermal growth factor receptor; EGFP, enhanced GFP; SBP, streptavidin binding protein; TGN, trans-Golgi network.

Next, we tested whether AP-1 interacts with EGFR using a streptavidin pull-down assay. HEK293T cells coexpressing HA-tagged *Escherichia coli* biotin ligase (HA-BirA) and EGFR bearing an EGFP and a biotin acceptor peptide (Bio tag) at its C-terminus (EGFR-EGFP-Bio) were harvested and lysed. The cell lysates were then incubated with streptavidin beads. The bound proteins were analyzed by immunoblotting. HEK293T cells coexpressing HA-BirA and EGFP with a Bio tag at its C-terminus (EGFP-Bio) were used as a negative control. We found that AP1γ1 interacted with EGFR, whereas epsinR, and the delta subunit of the adaptor complex-3 (AP3δ1) showed no binding (Fig. 2*A*). As an additional approach, we performed co-immunoprecipitation (co-IP) experiments and found that AP1γ1 but not ERGIC53 co-immunoprecipitated with HA- and FLAG-tagged EGFR (HA-EGFR-FLAG, Fig. 2*B*). By contrast, AP1γ1 did not co-immunoprecipitate with FLAG-tagged p75 (p75-FLAG, Fig. 2*C*), indicating that EGFR but not p75 interacts with AP1γ1.

Next, we investigated the motif on EGFR that binds to AP-1. Streptavidin pull-down assay was performed using EGFP-Bio-tagged full-length EGFR (EGFR^1-1210^-EGFR-Bio) or different EGFR fragments (Fig. 2*D*): an N-terminal fragment of EGFR containing the transmembrane domain (EGFR^1-668^-EGFP-Bio), a fragment containing the cytosolic domain (EGFR^669-1210^-EGFP-Bio), the EGFR kinase domain (EGFR^669-968^-EGFP-Bio), and the cytosolic tail of EGFR (EGFR^969-1210^-EGFP-Bio). The results indicate that AP1γ1 interacted with the full length, the cytosolic domain and the cytosolic tail of EGFR (Fig. 2*E*, lanes 1 and 4 and Fig. 2*G*). By contrast, the N-terminal fragment showed a weak interaction with AP1γ1 (Fig. 2*E*, lane 2 and Fig. 2*G*). EGFR^669-968^-GFP-Bio did not express well in HeLa cells (Fig. 2*E*, lane 7). These analyses indicate that the AP-1–binding motif is located within the EGFR cytosolic tail (969–1210).

The EGFR cytosolic tail contains a conserved tyrosine sorting motif: YRAL (998–1001) (Fig. 2*F*, highlighted in the red box). We then performed streptavidin pull-down experiments to test whether this motif is important for AP-1 binding. Consistent with our previous results, full-length EGFR interacted with AP1γ1 (Fig. 2*H*, lane 1), whereas the Y998A substitution greatly reduced the interaction between AP-1 and EGFR (Fig. 2*H*, compare lanes 1 and 2). EGFR bearing a mutation of a tyrosine residue at another site (EGFR^Y1172A^) did not cause a defect in binding to AP-1 (Fig. 2*H*, lane 3). This result suggests that the YRAL motif of EGFR is an AP-1–binding site on EGFR.

We then performed the RUSH assay to test whether Y998A substitution causes defects in TGN export of EGFR. The majority of WT SBP-EGFP-EGFR showed a cell-surface localization with no detectable EGFR at the juxtanuclear Golgi area 2 h after biotin treatment (Fig. 2*I*), whereas the majority of SBP-EGFP-EGFR^Y998A^ mutant was accumulated at the juxtanuclear Golgi area colocalized with the TGN marker, TGN46, at this condition (Fig. 2*I*). Quantification analysis indicated that the percentage of cells showing TGN-accumulated SBP-EGFP-EGFR^Y998A^ was significantly higher than that detected in cells expressing SBP-EGFP-EGFR^WT^ (Fig. 2*J*), indicating that the Y998 A mutation causes defects in TGN export of EGFR. These analyses suggest that AP-1 interacts with Y998 residue of EGFR to regulate its TGN export.

### Rab12 regulates TGN export of EGFR

**Figure 3:**
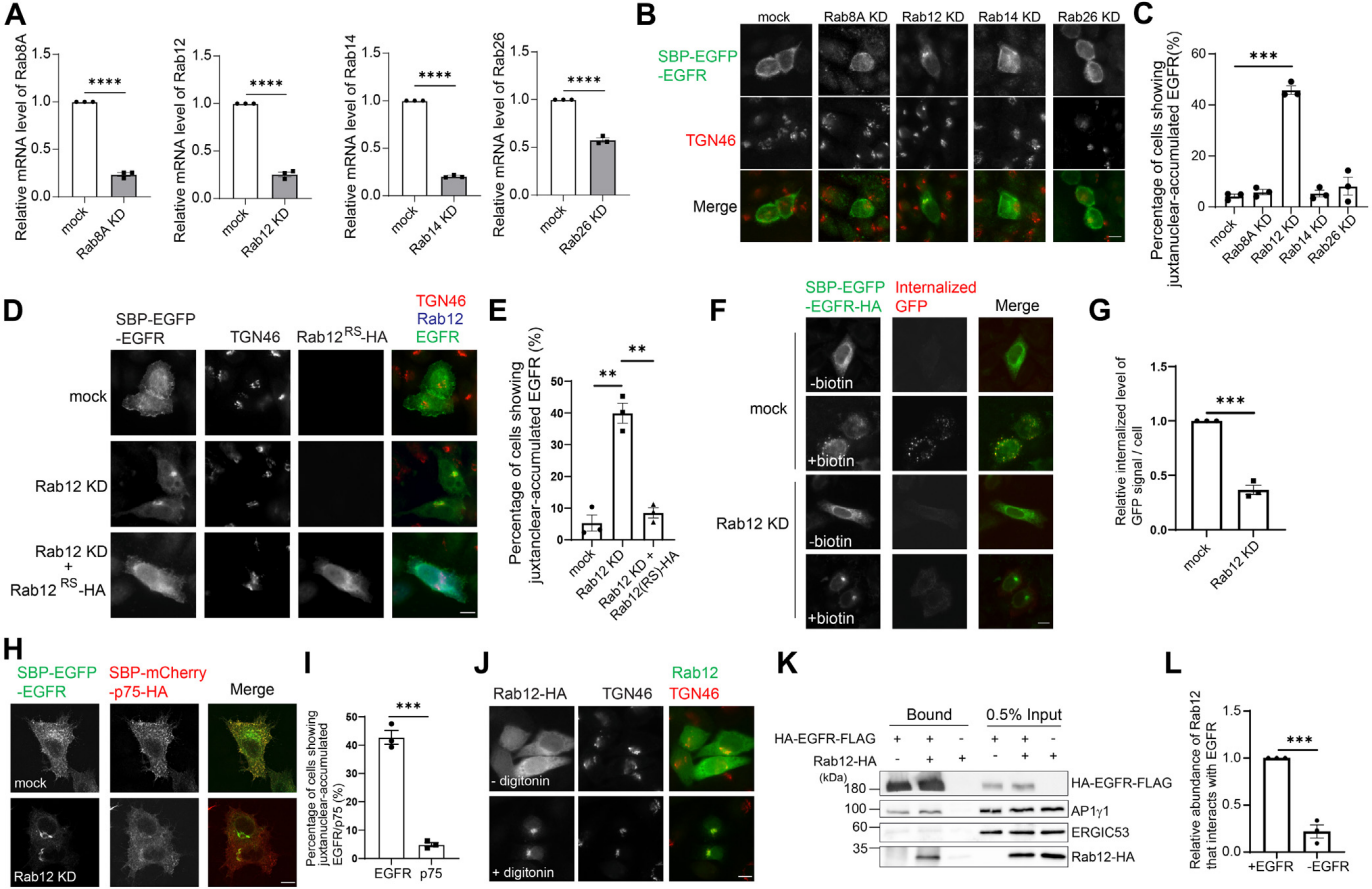
**Rab12 interacts with EGFR and regulates the TGN-to-cell surface delivery of EGFR.***A*, HeLa cells were mock transfected or transfected with siRNA against the indicated Rab proteins. Forty-eight hours after transfection, the relative mRNA levels of the indicated Rab proteins were analyzed by RT-qPCR (*n* = 3, mean ± SEM). *B* and *C*, HeLa cells were mock transfected or transfected with siRNA against the indicated Rab proteins and retransfected after 24 h with plasmid encoding SBP-EGFP-EGFR. After an additional 24 h, the localizations of the indicated proteins were analyzed by immunofluorescence (*B*). The size bar represents 10 μm. The percentage of cells showing juxtanuclear-accumulated EGFR at the Golgi area was quantified (*C, n* = 3, mean ± SEM, over 100 cells were quantified in each experimental group). *D*, HeLa cells were mock transfected or transfected with siRNA against Rab12 and retransfected after 24 h with plasmid encoding the indicated constructs. After an additional 24 h, cells were incubated with biotin for 2 h, and the localizations of the indicated proteins were analyzed by immunofluorescence. The size bar represents 10 μm. *E*, the percentage of cells showing juxtanuclear-accumulated EGFR at the Golgi area was quantified (*n* = 3, mean ± SEM, over 100 cells were quantified in each experimental group). *F* and *G*, HeLa cells were mock transfected or transfected with siRNA against Rab12 and retransfected after 24 h with plasmid encoding SBP-EGFP-EGFR-HA. After an additional 24 h, rabbit anti-GFP antibodies were added to the medium in the presence or absence of biotin for 2 h. The internalized GFP antibodies were then detected by secondary antibodies against rabbit conjugated with 568 fluorophore. The size bar represents 10 μm. The ratio of internalized above-threshold fluorescent level of GFP signal over the above-threshold fluorescent level of total SBP-EGFP-EGFR-HA signal per cell was quantified (*G*, *n* = 3, mean ± SEM, over 15 cells were quantified in each experimental group). The quantification was normalized to the mock group in each experiment. *H*, mock transfected HeLa cells or Rab12 KD HeLa cells coexpressing SBP-EGFP-EGFR and SBP-mCherry-p75-HA were incubated with biotin for 2 h, and the localizations of the indicated proteins were analyzed by immunofluorescence. The size bar represents 10 μm. *I*, the percentage of cells showing juxtanuclear-accumulated EGFR or p75 in *E* was quantified (*n* = 3, mean ± SEM, over 100 cells were quantified in each experimental group). *J*, HeLa cells were transiently transfected with plasmids encoding Rab12-HA. Day 1 after transfection, HeLa cells expressing Rab12-HA were incubated with or without digitonin and washed with PBS. The localizations of the indicated proteins were analyzed by immunofluorescence. The size bar represents10 μm. *K*, lysates from HEK293T cells expressing the indicated constructs were incubated with M2 agarose beads. The bound proteins were analyzed by immunoblotting. *L*, the ratio of bound Rab12-HA level over the Rab12-HA level in 0.5% input was quantified (*n* =3, mean ± SEM). The quantification was normalized to the plus EGFR and Rab12 group in each experiment. ∗∗*p* < 0.01; ∗∗∗*p* < 0.001; ∗∗∗∗*p* < 0.0001. EGFR, epidermal growth factor receptor; EGFP, enhanced GFP; qRT-qPCR, quantitative reverse transcription RT-PCR; SBP, streptavidin binding protein; TGN, trans-Golgi network.

As Rab proteins play important roles in vesicular trafficking (15), we tested whether Rab proteins regulate the TGN export of EGFR. UniProt predicted that seven Rab proteins, including Rab8A, Rab12, Rab14, Rab20, Rab26, Rab30, and Rab36, are located at the Golgi. We performed knockdown (KD) screening of these Rab proteins to test whether they regulate TGN export of EGFR. Quantitative RT-PCR analysis indicated that siRNA against four of these Rab proteins, Rab8A, Rab12, Rab14, and Rab26, effectively reduced the expression of their targets (Fig. 3*A*). Using the RUSH release assay, we found that the percentage of cells showing accumulation of SBP-EGFP-EGFR at the juxtanuclear area in Rab12 KD cells was significantly higher than that detected in control cells after biotin treatment (Fig. 3, *B* and *C*), whereas KD of three other Rab proteins did not cause significant defects. The juxtanuclear-accumulated SBP-EGFP-EGFR was colocalized with TGN46 (Fig. 3*B*).

Next, we performed the RUSH release assay in Rab12 KD HeLa cells expressing a rescue construct, which contains nonsense mutations at the siRNA targeting site and an HA tag at its C-terminus (Rab12^RS^-HA). We found that the expression of Rab12^RS^-HA rescued the defects of TGN export of EGFR in Rab12 KD cells (Fig. 3, *D* and *E*). In addition, we performed the antibody uptake assay and found that the total fluorescence labeled by anti-GFP antibodies in Rab12 KD cells was significantly lower than that in mock cells after biotin treatment (Fig. 3, *F* and *G*), suggesting Rab12 KD causes defects in surface delivery of EGFR. Consistently, live imaging analysis indicated that SBP-EGFP-EGFR-HA was accumulated in the juxtanuclear area with no detectable delivery to the cell surface in Rab12 KD HeLa cells (Movie S3). We then performed our analysis in HeLa cells coexpressing SBP-EGFP-EGFR and SBP-mCherry-p75-HA. We found that KD of Rab12 caused defects in TGN export of SBP-EGFP-EGFR but did not affect the surface delivery of SBP-mCherry-p75-HA in the co-expressing cells (Fig. 3, *H* and *I*), suggesting that TGN export of p75 is independent of Rab12.

We then analyzed the localization of Rab12 in HeLa cells. Rab12-HA showed a cytosolic localization (Fig. 3*J*). To test whether Rab12 associates with membranes of intracellular compartments, we treated cells with digitonin to disrupt the plasma membrane and subsequently washed away the cytosolic proteins. We found that Rab12-HA showed Golgi localization after digitonin treatment (Fig. 3*J*). Further analysis indicates that Rab12-HA co-immunoprecipitated with HA-EGFR-FLAG (Fig. 3, *K* and *L*). These data suggest that Rab12 interacts with EGFR and regulates the TGN export of EGFR. Adding a tag at the C-terminus of Rab12 affects geranylgeranyl addition on the C-terminal cysteine residue of Rab12. We then generated an N-terminal HA-tagged Rab12 (HA-Rab12). HA-Rab12 also showed a diffuse cytosolic pattern before digitonin treatment and a Golgi-localized pattern after digitonin treatment (Fig. S1). This result indicates that the N- and C-terminal HA-tagged Rab12 showed a similar location pattern. The C-terminal HA-tagged Rab12 rescued the defects of TGN export of SBP-EGFP-EGFR in Rab12 KD cells (Fig. 3, *D* and *E*), indicating that geranylgeranyl modification did not affect the roles of Rab12 in regulating TGN export of EGFR.

### AP-1 and Rab12 regulate EGFR signaling

**Figure 4:**
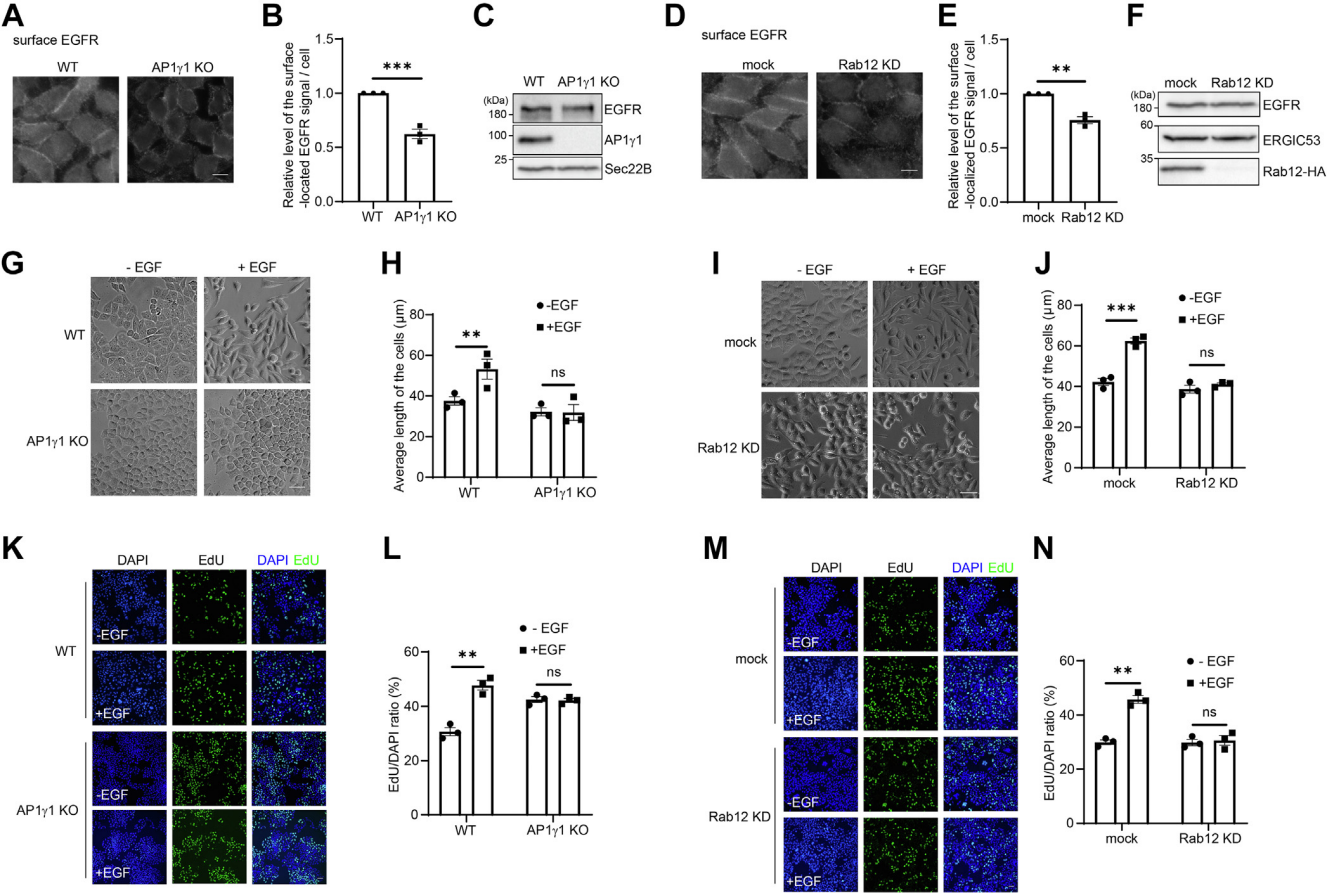
**AP-1 and Rab12 are important for EGFR signaling.***A* and *B* and *D*–*E*, EGFR surface labeling was performed in WT HeLa and AP1γ1 KO HeLa cells or in HeLa cells transfected with control siRNA or siRNA against Rab12. The size bar represents 10 μm. The relative level of the surface-located EGFR signal per cell was quantified (*n* = 3, mean ± SEM, over 100 cells were quantified in each experimental group). The quantification was normalized to the surface EGFR signal of WT HeLa cells (*B*) or mock-transfected HeLa cells (*E*) in each experimental group. *C* and *F*, the cell lysates of WT HeLa cells and AP1γ1 KO and Rab12 KD HeLa cells were analyzed by immunoblotting. An additional transfection of plasmids encoding Rab12-HA was performed in the mock and Rab12 KD cells to test the KD efficiency in panel *F*. *G*–*J*, WT HeLa and AP1γ1 KO HeLa cells (*G*) or HeLa cells transfected with control siRNA or siRNA against Rab12 (*I*) were incubated in medium with or without 10 ng/ml of EGF for 16 h. The length of the cells was quantified for each group (*n* = 3, mean ± SEM, over 100 cells were quantified in each experimental group). The length of the cells indicates the longest distance of two points at the cell surface. The size bar represents 50 μm. *K*–*N*, EdU proliferation assay was performed in WT HeLa and AP1γ1 KO HeLa cells (*K*) or in HeLa cells transfected with control siRNA or siRNA against Rab12 (*M*). The cells were incubated in medium with or without 10 ng/ml of EGF for 4 h and analyzed by immunofluorescence. The size bar represents 50 μm. The number of cells showing EdU signal was quantified and normalized to the number of cells showing DAPI signal (*n* = 3, mean ± SEM, over 300 cells were quantified in each experimental group). ∗∗*p* < 0.01; ∗∗∗*p* < 0.001. ns, not significant. AP-1, adaptor complex-1; DAPI, 4′,6-diamidino-2-phenylindole; EGF, epidermal growth factor; EGFR, epidermal growth factor receptor; EGFP, enhanced GFP; KD, knockdown.

Utilizing a surface labeling assay, we found that the knockout of AP1γ1 or the KD of Rab12 caused a significant reduction of the surface-located endogenous EGFR in HeLa cells (Fig. 4, *A*, *B* and *D*–*E*). By contrast, AP1γ1 KO or Rab12 KD did not cause a detectable change of the total level of endogenous EGFR in HeLa cells (Fig. 4, *C* and *F*). These results indicate that depletion of AP-1 or Rab12 affects the abundance of surface-located endogenous EGFR.

Given that the abundance of surface-located EGFR is a crucial determinant of the activity of EGF-induced EGFR signaling (16), we tested whether AP-1 and Rab12 regulate EGFR signaling. We first analyzed whether AP1γ1 KO or Rab12 KD affects EGF-induced phosphorylation of EGFR in HeLa cells. To test this, HeLa cells were starved for 24 h using medium without fetal bovine serum (FBS) before EGF treatment. Subsequently, cells were incubated in the starved medium in the presence of Baf A1 and EGF for 16 h. Baf A1 was added to inhibit the degradation of phosphorylated EGFR (pEGFR). We quantified the level of pEGFR and normalized the level of pEGFR to the total level of EGFR. The result showed that both AP1γ1 KO and Rab12 KD significantly reduced the normalized pEGFR level after EGF treatment (Fig. S2, *A* and *C*, quantifications in Fig. S2, *B* and *D*), suggesting that AP-1 and Rab12 are important for EGF-induced phosphorylation of EGFR. We noticed that the level of EGFR in AP1γ1 KO HeLa cells was lower than that in WT HeLa cells (Fig. S2*A*). We hypothesize AP1γ1 KO may affect the expression of EGFR under starved condition.

EGF-induced EGFR signaling causes accumulations of actin filaments in the cell periphery and elongations of cell shape (17, 18). Consistently, we found that WT HeLa cells changed into an elongated shape after 16 h of EGF treatment, whereas AP1γ1 KO HeLa or Rab12 KD cells showed no change in morphology (Fig. 4, *G*–*J*), suggesting that AP-1 and Rab12 are important for cell elongation induced by EGFR signaling. EGFR signaling also induces cell proliferation (19). To test whether AP1 and Rab12 are important for EGFR-induced cell proliferation, we performed the EdU proliferation assay. Cells were incubated in 50 mM EdU for 4 h in the presence or absence of 10 ng/ml of EGF. The total cells were visualized by 4′,6-diamidino-2-phenylindole (DAPI), and the newly replicated cells were visualized by EdU. Thus, we use the EdU/DAPI ratio to represent proliferation rate. We found that the proliferation was strongly promoted by EGF in WT HeLa cells but not in AP1γ1 KO HeLa or Rab12 KD cells (Fig. 4, *K*–*N*), indicating that AP-1 and Rab12 are crucial for the EGF-induced cell proliferation. Interestingly, we found that the proliferation rate of AP1γ1 KO HeLa cells is higher than that of WT HeLa cells (Fig. 4*L*), but the underlying mechanism is unknown. Taken together, these analyses suggest that AP-1 and Rab12 are important for the surface delivery of endogenous EGFR and regulate EGFR signaling.

### Surface delivery of EGFR^L858R^ is independent of AP-1 and Rab12

**Figure 5:**
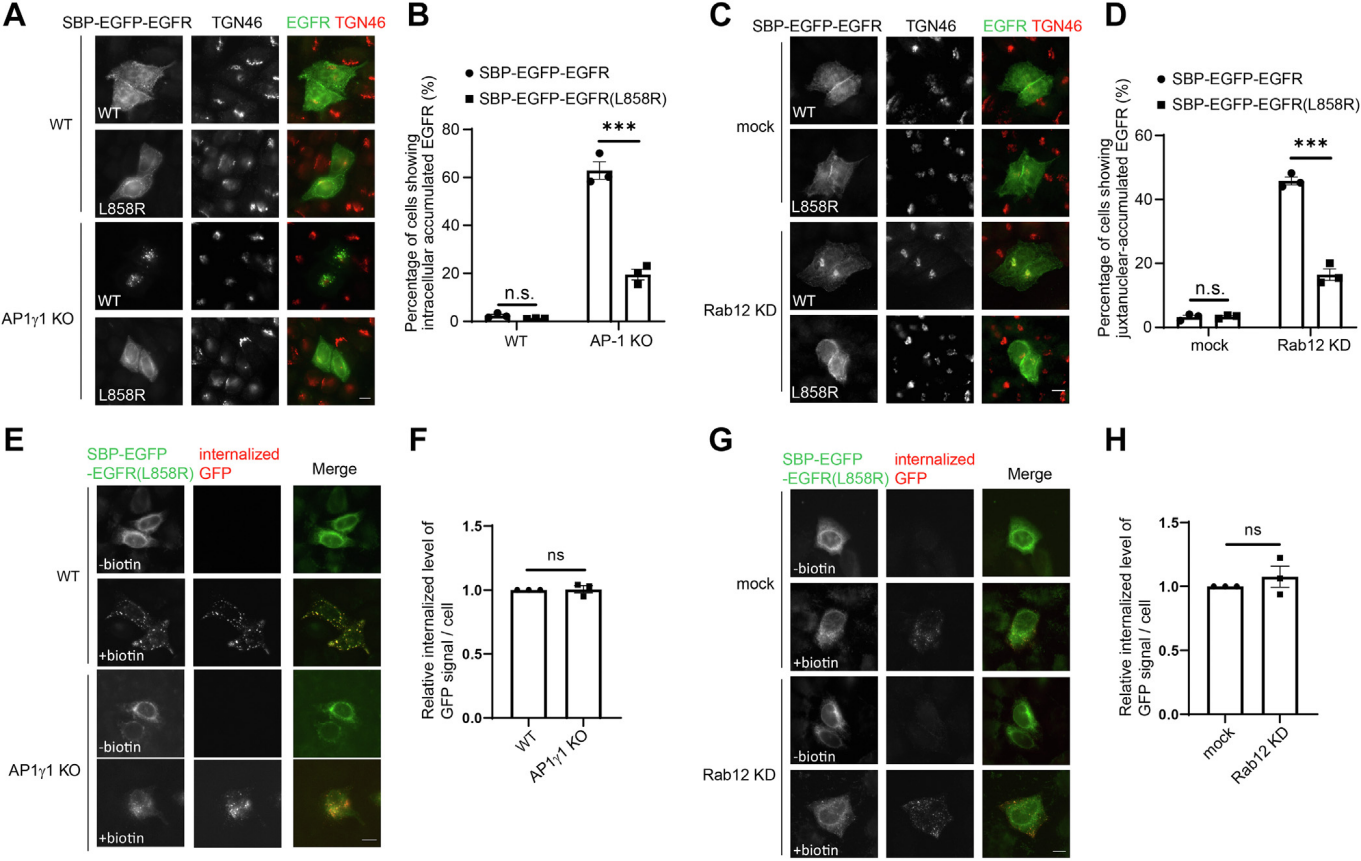
**Surface delivery of EGFR**^**L858R**^**is independent of AP-1 and Rab12.***A* and *C*, the localizations of the indicated proteins were analyzed by immunofluorescence in the indicated cells 2 h after biotin treatment. The size bar represents 10 μm. *B* and *D*, the percentage of cells showing intracellular accumulated EGFR (*B*) or juxtanuclear accumulated EGFR at the Golgi area (*D*) was quantified (*n* = 3, mean ± SEM, over 100 cells were quantified in each experimental group). *E* and *G*, antibody uptake assay was performed in WT HeLa cells or AP1γ1 KO HeLa cells or HeLa cells transfected with control siRNA or siRNA against Rab12. The level of internalized GFP antibodies and the localization of SBP-EGFP-EGFR^L858R^ were analyzed. The size bar represents 10 μm. *F* and *H*, the ratio of internalized above-threshold fluorescent level of GFP signal over the above-threshold fluorescent level of the total SBP-EGFP-EGFR^L858R^ signal per cell was quantified (*n* = 3, mean ± SEM, over 15 cells were quantified in each experimental group). The quantification was normalized to WT group or to the mock group in each experiment. ∗∗∗*p* < 0.001; ns, not significant. AP-1, adaptor complex-1; EGFP, enhanced GFP; EGFR, epidermal growth factor receptor; KD, knockdown; SBP, streptavidin binding protein.

In non-small cell lung cancer patients bearing an EGFR mutation, 45% of EGFR mutants bear an L858R mutation (8). We analyzed the kinetics of trafficking of WT EGFR and EGFR (L858R) using the RUSH assay by quantifying the percentage of cells showing accumulations of EGFR at the juxtanuclear Golgi area at different time points after biotin treatment (Fig. S3, *A* and *B*). This analysis indicates that the RUSH construct of WT EGFR and the EGFR (L858R) showed similar kinetics of trafficking in the secretory pathway. Given that EGFR^L858R^ mutant is constitutively phosphorylated, we hypothesized that EGFR^L858R^ is recognized by different cargo sorting machineries to mediate its TGN export process. Consistent with this hypothesis, we found that the knockout of AP1γ1 or the KD of Rab12 did not cause defects in the TGN-to-cell surface delivery of SBP-EGFP-EGFR^L858R^ in the RUSH assay (Fig. 5, *A*–*D*). The GFP antibody uptake assay also indicated that EGFR^L858R^ was transported to the surface of AP1γ1 KO HeLa cells and Rab12 KD HeLa cells with an efficiency that is similar to that detected in control cells (Fig. 5, *E*–*H*). These data suggest that surface delivery of EGFR^L858R^ is independent of AP-1 and Rab12.

## Discussion

**Figure 6:**
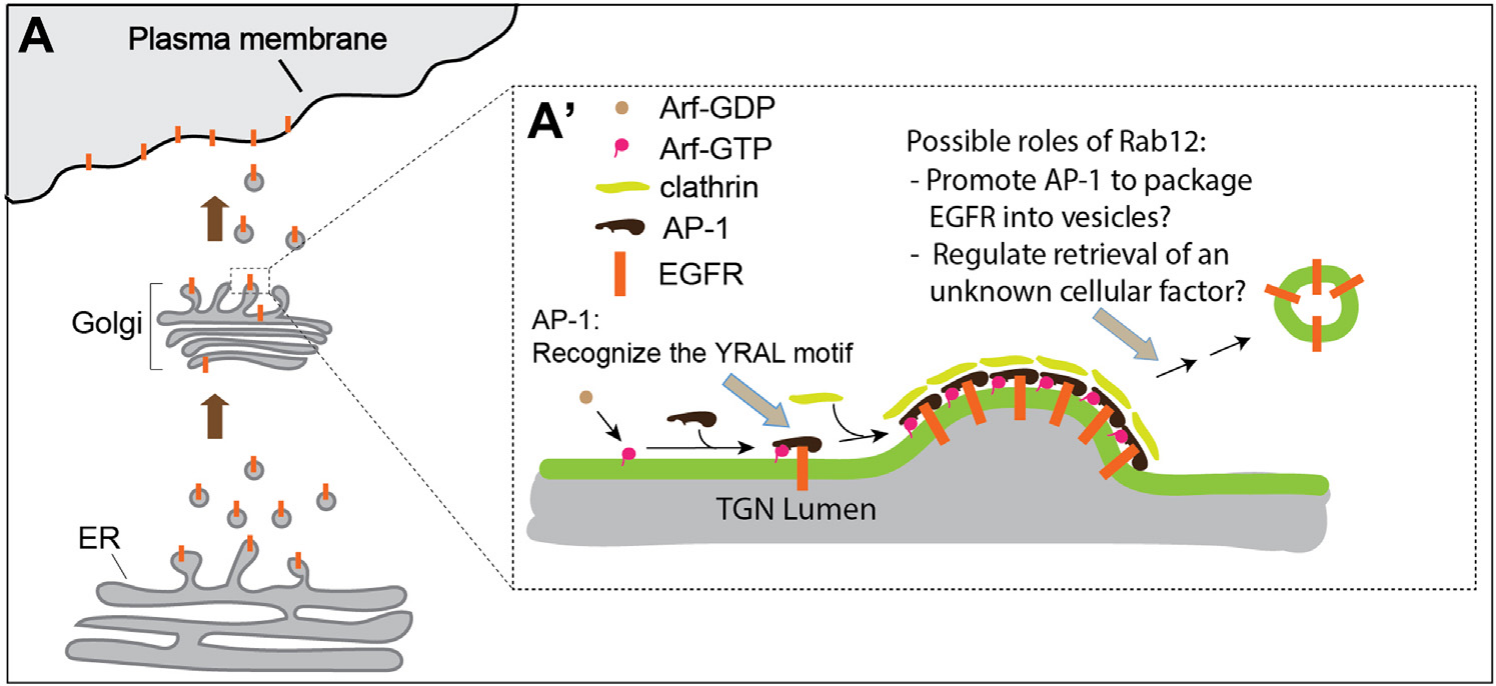
**A hypothesized model of EGFR post-Golgi trafficking.** AP-1 recognizes the YARL motif in EGFR to enrich EGFR into TGN-derived vesicles destined for the plasma membrane. Rab12 regulates the TGN export of EGFR by either directly promoting the TGN export process or indirectly regulating the trafficking of EGFR by mediating the retrograde transport of an unknown cellular factor that is critical for TGN export of EGFR. AP-1, adaptor complex-1; EGFR, epidermal growth factor receptor; TGN, trans-Golgi network.

The adaptor protein complexes recognize cargo proteins bearing particular motifs, including tyrosine-based sorting motifs (YXXΦ, where Φ represents an amino acid containing a bulky hydrophobic side chain) and dileucine sorting motifs ([DE]XXXL[LI]) (4). These motifs are important for the targeting of cargo proteins to endosomes, lysosomes, the basolateral surface of polarized epithelial cells, and the somatodendritic domain of hippocampal neurons (4, 20, 21, 22). Here, we identified a tyrosine sorting motif (Y^998^RAL) on EGFR that interacts with AP-1. The interaction between AP-1 and the Y^998^RAL motif on EGFR is crucial for the trafficking of EGFR from the TGN to the plasma membrane. We proposed that AP-1 recognizes the Y^998^RAL motif of EGFR, thereby promoting the enrichment of EGFR into TGN-derived vesicles destined for the plasma membrane (Fig. 6).

The Y^998^RAL motif of EGFR has been implicated to interact with the μ2 subunit of AP-2 (23). AP-2 also interacts with a dileucine motif (L^1034^/L^1035^) on EGFR (24). The clathrin-dependent endocytosis of activated EGFR is regulated by several redundant and interdependent mechanisms (25). The binding of AP-2 to the Y^998^RAL and L^1034^/L^1035^ motif is one of these mechanisms that contribute to EGFR endocytosis (25). The tyrosine sorting motif directly binds to the μ subunit of the adaptor complex. In addition to the conventional motif, an unconventional tyrosine sorting motif (YX[FYL][FL]E) from Alzheimer’s disease-associated amyloid precursor protein directly interacts with the μ subunit of AP-4 at a binding site that is opposite to the conventional tyrosine–motif-binding site (26). The key residues involved in binding to this unconventional motif are conserved in AP-1 (4). Whether the Y^998^RAL motif in EGFR binds the conventional tyrosine-binding site or other sites on AP-1 remains to be further investigated.

We noticed that EGFR is accumulated in LAMP2-postive structures in AP1γ1 KO cells, whereas mutating the Y^998^RAL motif accumulates EGFR at the TGN area. A possible explanation for this observation is that EGFR followed two trafficking routes upon exiting the TGN: one route destined to the cell surface and another route destined to lysosomes for degradation. In WT HeLa cells, EGFR was mainly delivered along the route to the cell surface mediated by AP-1. In AP1γ1 KO cells, EGFR was delivered along the second route to lysosomes by another cargo sorting machinery, and this sorting machinery also recognizes the tyrosine sorting motif on EGFR. In this scenario, mutating the tyrosine sorting motif in EGFR not only blocks the surface delivery of EGFR but also blocks the delivery of EGFR from the TGN to lysosomes, thereby causing accumulations of EGFR at the TGN.

The Rab GTPases mediate the budding, transport, docking, and fusion of transport vesicles (15). Rab12 is one of the less well-characterized Rab family proteins and has been shown to mediate trafficking in the endocytic pathway. Rab12 is important for the trafficking of internalized transferrin receptors from recycling endosomes to lysosomes for degradation (27), and it regulates the retrograde transport of the B-subunit of Shiga toxin from the plasma membrane to the TGN (28). Rab12 interacts with the Rab7-interacting lysosomal protein–dynein complex (29) and regulates the retrograde transport of secretory granules in activated mast cells (30). In addition, Rab12 promotes the degradation of proton-coupled amino-acid transporter 4 to regulate mTORC1 signaling and autophagy (31). In this study, we found that Rab12 localized to the Golgi and regulated TGN-to-cell surface delivery of newly synthesized EGFR, thereby regulating the surface level of EGFR. We demonstrated that this step is critical for EGF-induced EGFR signaling. These analyses indicate that Rab12 not only regulates endocytic trafficking but also regulates trafficking in the secretory pathway. We hypothesized that Rab12 may promote AP-1 to recognize EGFR to enrich EGFR into TGN-derived vesicles. Rab12 may also be important for the retrograde transport of an unknown cellular factor from endosomes to the TGN to mediate the TGN export of EGFR (Fig. 6).

The activation of WT EGFR is initiated by the ligand-induced dimerization of the EGFR extracellular domain, which directs asymmetric dimerization of the N- and C-lobes in the EGFR cytosolic kinase domain (32). The constitutive activation of various EGFR oncogenic mutants is not dependent on ligand binding, and two distinct requirements of dimerization for oncogenic activation were shown to exist among these mutants (33). Mutants including EGFR^L858R^ depend on dimerization for constitutive receptor activation, while mutants including Ex 19Del do not require dimerization for their oncogenic activation (33). In contrast to WT EGFR, EGFR^L858R^ is able to form stable, ligand-independent dimers with a more extended conformation (10, 34). The dimerization of EGFR has also been shown to induce a conformational change of its cytosolic domain (32, 35). We proposed that the conformational changes in the cytosolic domain and the luminal domain of EGFR induced by L858R mutation expose new binding sites for other cellular factors which promote TGN export of EGFR independent of Rab12 or AP-1. Uncovering the cellular factors that are important for TGN export of EGFR^L858R^ will further elucidate the differences between the molecular machineries regulating the trafficking of WT EGFR and EGFR^L858R^.

## Experimental procedures

### Cell lines, antibodies, plasmids, immunofluorescence, and transfection

HeLa cells and HEK293T cell lines were kindly provided by the University of California-Berkeley Cell Culture Facility and were confirmed by short tandem repeat profiling. AP1γ1 KO HeLa was generated as described (36). All cell lines were tested negative for *Mycoplasma* contamination and cultured in Dulbecco's Modified Eagle Medium (DMEM) containing 10% FBS and 1% Penicillin-Streptomycin mix (Thermo Fisher Scientific, #15140122).

The commercial antibodies were rabbit anti-HA (Cell Signaling #3724, RRID: AB_1549585), mouse anti-HA (Biolegend, catalogue number 901501), sheep anti-TGN46 (AbD Serotec, number AHP500G, RRID: AB_323104), mouse anti-FLAG (Sigma-Aldrich, number F3165, RRID: AB_259529), mouse anti-LAMP2 (Developmental Studies Hybridoma Bank, #H4B4), mouse anti-γ1 subunit of AP-1 (BD Bio #610385, RRID: AB_397768), mouse anti-δ subunit of AP-3 (Developmental Studies Hybridoma Bank, number anti-delta SA4, RRID: AB_2056641), rabbit anti-epsinR (Bethyl Laboratories, A301–926A), and mouse anti-EGFR (Santa Cruz Biotechnology, #sc-101). Rabbit anti-Sec22B, rabbit anti-Sec23A, and rabbit anti-ERGIC53 antibodies were kindly provided by Prof. Randy Schekman (University of California). Rabbit anti-GFP antibodies were kindly provided by Prof. Robert Qi (Hong Kong University of Science and Technology).

The RUSH constructs of EGFR (SBP-EGFP-EGFR or SBP-EGFP-EGFR-HA) and the RUSH construct of p75 (SBP-mCherry-p75-HA) were generated by replacing DNA encoding Ecadherin from Str-KDEL_SBP-EGFP-Ecadherin (Addgene, Plasmid #65286) or Str-KDEL_SBP-mCherry-Ecadherin (Addgene, Plasmid #65287) with DNA encoding EGFR (31–1210), EGFR (31–1210)-HA, or p75 (29–427). Plasmids encoding EGFP-Tev-Bio and plasmids encoding HA-BirA were gifts from Prof. Robert Qi (Hong Kong University of Science and Technology). EGFR-EGFP-Bio was generated by cloning full-length EGFR into the vector encoding EGFP-Tev-Bio. Different EGFR truncations: EGFR (669–1210)-EGFP-Bio, EGFR (1–668)-EGFP-Bio, EGFR (1–968)-EGFP-Bio, EGFR (669–698)-EGFP-Bio, and EGFR (969–1210)-EGFP-Bio were generated by standard molecular cloning procedures. Different EGFR mutations: EGFR (Y998A)-EGFP-Bio, EGFR (Y1172A)-EGFP-Bio, Str-KDEL_SBP-EGFP-EGFR (Y998A), and Str-KDEL_SBP-EGFP-EGFR (L858R) were generated by QuikChange II site-directed mutagenesis. Rab12-HA, Rab12^RS-^HA, and HA-EGFR-FLAG (a single HA tag is inserted following the signal peptide) were synthesized by BGI in pcDNA3.1 vector. HA-tagged AP1γ1 was generated by replacing DNA encoding Rab12 from Rab12-HA with DNA encoding AP1γ1. p75-FLAG was generated by cloning p75 into the p3xFLAG-CMV-14 (Sigma-Aldrich) vector. All of the DNA constructs were verified by sequencing.

The target sequence of siRNA against Rab12 is GAATGAGTTGTCCAATAGT. The target sequence of siRNA against Rab8A is GCATCATGCTGGTCTACGA. The target sequence of siRNA against Rab14 is CCATACAACTACTCTTACA. The target sequence of siRNA against Rab26 is GCTTCCGGCTGCATGATTA.

Transfection of siRNA or DNA constructs into HeLa cells, HEK293T cells, or AP1γ1 KO HeLa cells and immunofluorescence staining were performed as described previously (37). To measure the efficiency of KD of Rab12, HeLa cells were transfected with control siRNA or siRNA against Rab12. Day 1 after transfection, the cells were retransfected with plasmids encoding Rab12-HA. Day 3 after siRNA transfection, the cell lysates were analyzed by immunoblotting. Images were acquired with a Zeiss Axio Observer Z1 microscope system (Carl Zeiss AG) equipped with an ORCA Flash 4.0 camera (Hamamatsu).

### RUSH release assay, digitonin treatment, and EGF treatment

To perform the RUSH release assay, cells were first transfected with RUSH plasmid for 24 h. Then, the cells were treated with 100 ng/μl of cycloheximide (Sigma-Aldrich) for 2 h to stop the protein synthesis. To release the cargo from the ER, the cells were treated with 40 μM D-Biotin (Sigma-Aldrich) and 100 ng/μl of cycloheximide for the indicated time. Afterward, the immunofluorescence staining was performed.

To perform the live cell confocal imaging of the RUSH construct of EGFR, mock, AP1γ1 KO, or Rab12 KD HeLa cells seeded in a confocal glass dish were transfected with SBP-EGFP-EGFR-HA for 24 h. After treatment with 100 ng/μl of cycloheximide for 2 h, the cells were incubated in medium containing 40 μM of D-Biotin and 100 ng/μl of cycloheximide and ready for the live cell confocal imaging. The live imaging was performed using a Leica SP8 Confocal Microscope with a sample holder heated to 37 °C using a 63× objective camera. The images were taken under a xyzt projection with a 1-min interval. It took time to set up the imaging condition after adding biotin, so the starting time point of each movie was not immediately after adding biotin. The multiple z stacks were merged by ImageJ (fiji-win64) in maximum intensity projection.

The digitonin treatment was performed as described previously (38). The cells on the coverslip were washed with 1× KOAc buffer (110 mM potassium acetate, 20 mM Hepes, pH 7.2, 2 mM magnesium acetate) for two times. Then, the cells were permeabilized by incubation with 1× KOAc buffer containing 40 μg/ml of digitonin on ice for 5 min. Afterward, a wash with 1× KOAC buffer, a 5-min incubation with 5× KOAc buffer, and a final wash with 1× KOAC buffer were performed to wash away the cell cytosol. Finally, the immunofluorescence staining was performed.

The EGF treatment to test the morphology change of the cells was performed by incubating the cells in DMEM plus 10% FBS with 10 ng/ml of EGF (Sigma-Aldrich) for 16 h. The EGF treatment to test the pEGFR abundance was performed by starving the cells in DMEM without FBS for 24 h. The cells were then incubated in starvation medium with 100 nM of Baf A1 (Sigma-Aldrich) in the presence or absence of 10 ng/ml of EGF for 16 h. The cell lysates were then analyzed by immunoblotting.

### Surface labeling and antibody uptake assay

To perform the surface labeling of EGFR in mock, Rab12 KD, AP1γ1 KO, or WT HeLa cells, mouse anti-EGFR antibody (Santa Cruz, #sc-101) was used to label the extracellular domain of EGFR. HeLa cells were washed with cold PBS for five times and incubated with mouse anti-EGFR antibodies (1: 200) in PBS containing 2.5% FBS (250 μl in 10 ml) for 40 min on ice. After several washes with PBS, cells were fixed for 15 min with 4% paraformaldehyde in PBS, and then the immunofluorescence staining was performed.

To perform the antibody uptake assay, rabbit anti-GFP antibodies were used to label the EGFP tag in the extracellular side of SBP-EGFP-EGFR-HA construct. After treatment with 100 ng/μl of cycloheximide for 2 h, the cells expressing SBP-EGFP-EGFR-HA were incubated in medium containing 40 μM D-Biotin, 100 ng/μl of cycloheximide, and rabbit anti-GFP antibodies (1:200) at 37 °C for 2 h. After several washes with PBS, the cells were fixed for 15 min with 4% paraformaldehyde in PBS, and then the immunofluorescence staining was performed. The above-threshold fluorescent level of internalized anti-GFP antibodies and total SBP-EGFP-EGFR-HA signal were measured by ImageJ (fiji-win64) after setting the same threshold.

### Streptavidin pull-down and co-IP

HEK293T cells expressing FLAG-tagged proteins were used to perform FLAG co-IP. HEK293T cells coexpressing GFP-Tev-Bio-tagged protein and HA-BirA were used to perform the streptavidin pull-down. Cells were lysed by lysis buffer (110 mM potassium acetate, 20 mM Hepes, pH 7.2, 2 mM magnesium acetate, 0.5% Triton X-100, 1 mM dithiothreitol, and 1× cOmplete Protease Inhibitor Cocktail (Roche Holding AG)) for 30 min on ice. The lysate was then centrifuged at 14,000*g* for 5 min at 4 °C, and the supernatant was incubated with beads prewashed with 1× KOAc buffer (110 mM potassium acetate, 20 mM Hepes, pH 7.2, 2 mM magnesium acetate) overnight at 4 °C with rotation. Dynabeads M-280 Streptavidin (Invitrogen) were used to pull down GFP-Tev-Bio-tagged proteins from cell lysates. Anti-FLAG M2-agarose affinity beads were used to immunoprecipitate FLAG-tagged proteins from cell lysates. After incubation, the beads were washed three times with 1× KOAc buffer and incubated with 2× protein sample buffer (100 mM Tris–HCl pH 6.8, 0.2% bromophenol blue, 4% SDS, 20% glycerol, and 25% β-mercaptoethanol) at 55 °C for 30 min for Western blot (WB) analysis. The M2 beads were incubated in elution buffer (0.6 mg/ml FLAG peptides in 1× KOAc buffer containing 1 mM dithiothreitol and 1× cOmplete Protease Inhibitor Cocktail) overnight at 4 °C with rotation. The eluted proteins were analyzed by WB. The intensity of the WB bands was measured using ImageJ after subtracting the background.

## RNA isolation and quantitative RT-PCR

Total RNA was extracted from the cultured cells using TRIzol reagent (Invitrogen), according to the manufacturer’s instructions. Reverse transcription was performed using high-capacity cDNA Reverse Transcription Kits (Applied Biosystems, #4368813). The cDNA determination was performed using TB Green Premix Ex Taq II (TaKaRa Bio Inc, #RR820A). Three technical repeats were performed in each experiment. After the reactions were completed, the cycle threshold (CT) data were determined using fixed threshold settings, and the mean CT was determined from triplicate PCRs. The mRNA levels were normalized to β-actin. The relative abundance of gene normalized to control was calculated with the equation 2^-ΔCT^, in which ΔCT=CT gene - CT control.

Primers of Rab12 were 5′-CTGGGATGCGGTTCTGTGAAG-3′ (sense), 5′-CAGTTCTGGCGGTATCTCAGG-3′ (anti-sense).

Primers of Rab8A were 5′-CAACGGCCTACTACAGGGG-3′ (sense), 5′-GGATGTTGTCGAAGGACTTCTC-3′ (anti-sense).

Primers of Rab14 were 5′-TATGGCTGATTGTCCTCACACA-3′ (sense), 5′-CTGTCCTGCCGTATCCCAAAT-3′ (anti-sense).

Primers of Rab26 were 5′-GTCTGCTGGTGCGATTCAAG-3′ (sense), 5′-GCATGGGTAACACTGCGGA-3′ (anti-sense).

Primers of β-actin were 5′-CATGTACGTTGCTATCCAGGC-3′ (sense), 5′-CTCCTTAATGTCACGCACGAT-3′ (anti-sense).

### EdU proliferation assay

The EdU Cell Proliferation Kit (Thermo Fisher Scientific, #C10337) was used to visualize the newly replicated cells. Cells were incubated in 50 mM EdU for 4 h under growth conditions with or without 10 ng/ml of EGF. After PBS wash, the cells were fixed with 4% paraformaldehyde for 15 min at room temperature (RT) and washed with PBS for five times. Then, the cells were incubated in permeabilization and blocking buffer (2.5% FBS, 0.1% TX100, 0.2 M Glycine in PBS) for 30 min at RT. Finally, the cells were incubated in EdU reaction solution (Thermo Fisher Scientific, #C10337) for 30 min at RT protected from light and washed with PBS for five times. DAPI was visualized using ProLong Gold Antifade Mountant (Invitrogen, # P36930).

## Data availability

All data are contained within the article and accompanying supporting information.

## Supporting information

This article contains supporting information.

## Conflict of interest

The authors declare that they have no conflicts of interest with the contents of this article.
