# Supplementary material for: The clathrin adaptor complex-1 and Rab12 regulate post-golgi trafficking of WT epidermal growth factor receptor (EGFR)
Source: J Biol Chem. 2023 Feb 4;299(3):102979. doi: 10.1016/j.jbc.2023.102979 (PMC10017364; doi:10.1016/j.jbc.2023.102979)
Supplement: Supporting information [file mmc1.pdf]

Figure S1

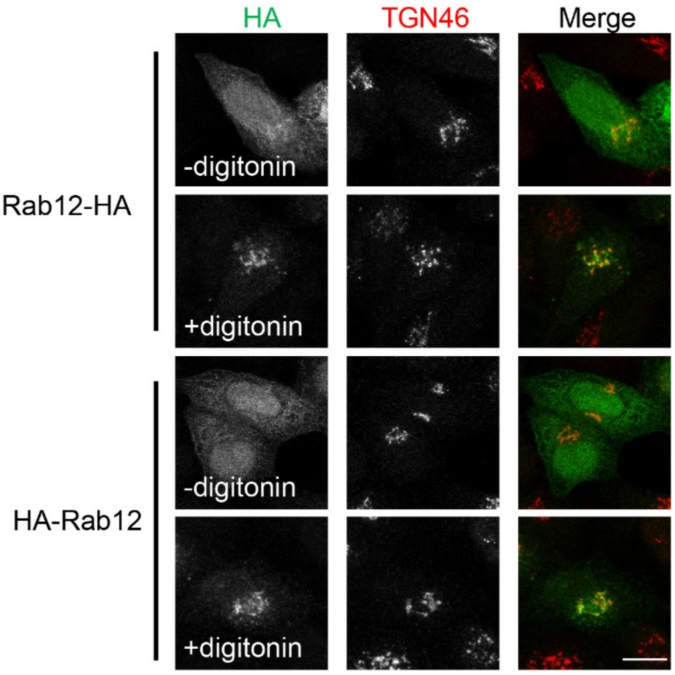

Figure S1. N-terminal and C-terminal HA-tagged Rab12 are located at the TGN. HeLa cells expressing Rab12-HA or HA-Rab12 were incubated with or without 40 µg/mL digitonin for 5 min on ice and washed with PBS. The localizations of the indicated proteins were analyzed by immunofluorescence. Size bar, 10 µm.

Figure S2

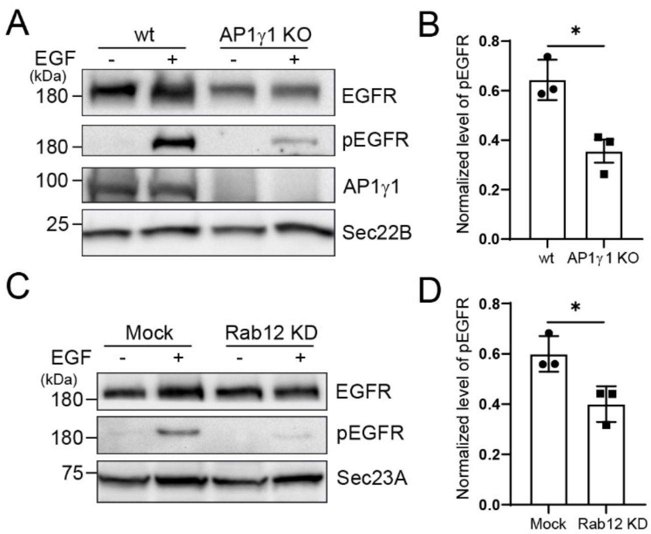

Figure S2. AP1γ1 KO or Rab12 KD causes defects in EGF-induced EGFR phosphorylation. *A and C,* wt HeLa and AP1γ1 KO HeLa cells (*A*) or HeLa cells transfected with control siRNA or siRNA against RAB12 (*C*) were starved in medium without FBS for 24h. The cells were then incubated in starvation medium with or without 10 ng/mL of EGF and 100 nM of Baf A1 for 16 h. The cell lysates were analyzed by immunoblotting. *B and D*, The ratio of the abundance of pEGFR over the abundance of total EGFR level in plus EGF group was quantified (n =3, mean ± SD). The quantification was normalized to the sum of value in the wt and AP1γ1 KO HeLa group (*B*) or the sum of the value in the Mock and Rab12 KD group (*D*) in each experiment. *, $p<0.05$.

Figure S3

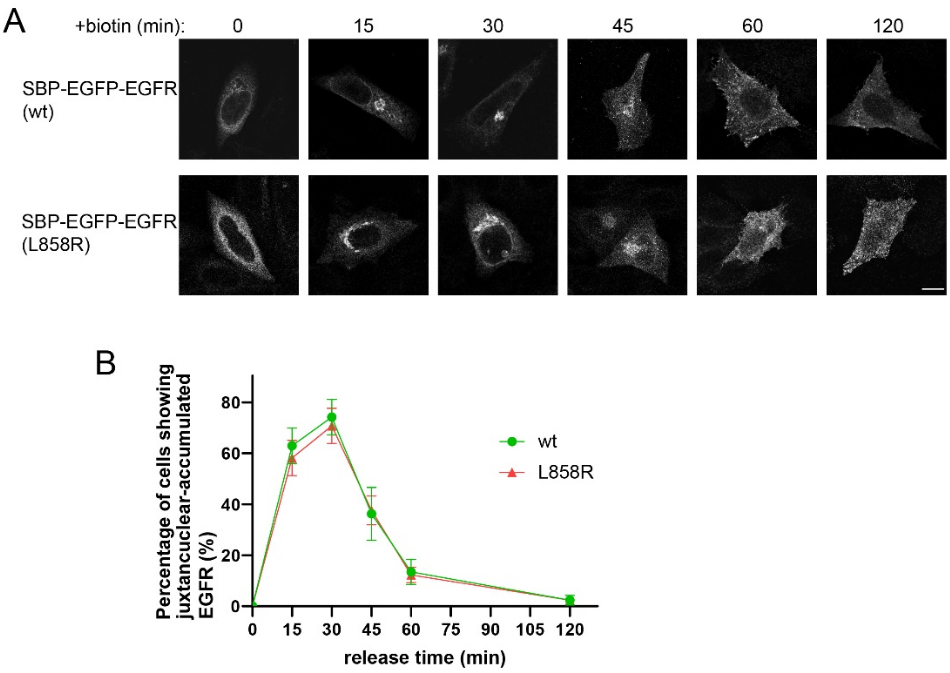

Figure S3. The kinetics of trafficking of SBP-EGFP-EGFR(L858R) along the secretory pathway is similar to SBP-EGFP-EGFR (wt). *A*, HeLa cells expressing SBP-EGFP-EGFR (wt) or SBP-EGFP--EGFR(L858R) were incubated with biotin for the indicated time, and the localizations of SBP-EGFP-EGFR (wt) or SBP-EGFP-EGFR(L858R) were analyzed by immunofluorescence. Size bar, 10 µm. *B*, The percentage of cells showing juxtanuclear-accumulated EGFR or EGFR(L858R) was quantified (n = 3, mean ± SD, over 100 cells were quantified in each experimental group)
